# Recent advances in the Design-Build-Test-Learn (DBTL) cycle for systems metabolic engineering of *Corynebacterium glutamicum*

**DOI:** 10.71150/jm.2501021

**Published:** 2025-03-28

**Authors:** Subeen Jeon, Yu Jung Sohn, Haeyoung Lee, Ji Young Park, Dojin Kim, Eun Seo Lee, Si Jae Park

**Affiliations:** Department of Chemical Engineering and Materials Science, Graduate Program in System Health Science and Engineering, Ewha Womans University, Seoul 03760, Republic of Korea

**Keywords:** Design-Build-Test-Learn (DBTL) cycle, metabolic engineering, systems metabolic engineering, *Corynebacterium glutamicum*, L-lysine, C5 chemicals

## Abstract

Existing microbial engineering strategies—encompassing metabolic engineering, systems biology, and systems metabolic engineering—have significantly enhanced the potential of microbial cell factories as sustainable alternatives to the petrochemical industry by optimizing metabolic pathways. Recently, systems metabolic engineering, which integrates tools from synthetic biology, enzyme engineering, omics technology, and evolutionary engineering, has been successfully developed. By leveraging modern engineering strategies within the Design-Build-Test-Learn (DBTL) cycle framework, these advancements have revolutionized the biosynthesis of valuable compounds. This review highlights recent progress in the metabolic engineering of *Corynebacterium glutamicum*, a versatile microbial platform, achieved through various approaches from traditional metabolic engineering to advanced systems metabolic engineering, all within the DBTL cycle. A particular focus is placed C5 platform chemicals derived from L-lysine, one of the key amino acid production pathways of *C. glutamicum*. The development of DBTL cycle-based metabolic engineering strategies for this process is discussed.

## Introduction

The urgent challenge of environmental pollution requires a global effort to mitigate its impact, counteract the accelerating effects of global warming, and develop sustainable alternatives to petrochemical industries. A comprehensive approach is crucial for addressing these environmental crises, facilitating the transition to eco-friendly practices, and promoting a circular economy that prioritizes renewable resources. In this context, metabolic engineering presents a promising solution by enabling the development of highly efficient microbial cell factories that utilize renewable resources as inputs to produce value-added products such as specialty chemicals and biopolymers. This innovation paves the way for a more sustainable and environmentally responsible industrial future (Hwang et al., 2024; Jung et al., 2024; Kim et al., 2024; Lee et al., 2024).

Traditional metabolic engineering focuses on optimizing metabolic pathways through strategies such as gene overexpression, targeted gene deletions, and the introduction of heterologous genes. These approaches aim to enhance the production of desired metabolites by modifying cellular processes. Over time, advancements in scientific knowledge and technological innovation have propelled the field forward, leading to the development of more sophisticated methodologies. Modern metabolic engineering now integrates cutting-edge technologies such as systems biology, synthetic biology, and evolutionary engineering, collectively forming the advanced framework of systems metabolic engineering. Systems biology provides a holistic understanding of cellular networks and their interactions, enabling the precise identification of metabolic bottlenecks and regulatory nodes. Synthetic biology introduces tools for designing and constructing novel biological circuits and pathways with unprecedented precision; evolutionary engineering applies selective pressure to develop strains with enhanced traits through adaptation processes that mimic natural evolution. Together, these approaches synergistically refine traditional metabolic engineering, enabling the development of microbial cell factories with significantly improved efficiency, robustness, and versatility for producing a wide range of value-added bioproducts (Chae et al., 2017; Choi et al., 2019; Desai et al., 2024; Gupta et al., 2024; Lee et al., 2011, 2012, 2019; Son et al., 2023).

*Corynebacterium glutamicum*, originally discovered for its ability to produce amino acids, has become a valuable organism in biotechnology. One of its key advantages in metabolic engineering is that its complete genome sequence was analyzed relatively early among industrially relevant host strains, making it particularly amenable to metabolic remodeling through genomic engineering techniques (Bendt et al., 2003). This comprehensive genomic knowledge enables researchers to make precise modifications to its metabolic pathways, enhancing its ability to produce a wide range of metabolites. A prime example of the metabolic potential of *C. glutamicum* is its production of L-lysine, a major feed additive. The successful large-scale fermentation of metabolically engineered *C. glutamicum* strains for L-lysine production not only demonstrates a major achievement in metabolic engineering but also establishes *C. glutamicum* as a highly capable platform strain. As a result, extensive research has been conducted to expand its application to a diverse range of metabolic products. To further establish *C. glutamicum* as an efficient microbial cell factory, its use has extended beyond the traditional production of amino acids and organic acids to the biosynthesis of high-value specialty chemicals (Han et al., 2020; Han & Lee, 2023; Rohles et al., 2018, 2022; Sohn et al., 2021, 2022, 2024; Wen et al., 2024). This progress has been driven by advanced engineering techniques, significantly improving strain engineering and adaptive evolution capabilities. These advancements allow for precise metabolic fine-tuning, enhanced stress tolerance, and optimized production pathways, making *C. glutamicum* a versatile and robust platform for sustainable bioprocessing.

In this review, we examine traditional and systems metabolic engineering strategies applied to *C. glutamicum*, with a particular focus on the Design-Build-Test-Learn (DBTL) cycle framework. This systematic and iterative approach facilitates the design, construction, testing, and refinement of genetically modified strains (Chen et al., 2017; Zhao et al., 2024). Advanced systems metabolic engineering technologies have not only enhanced the manufacturing of traditional amino acids in *C. glutamicum* but have also propelled modern metabolic engineering forward, enabling the precise optimization of metabolic pathways and the biosynthesis of innovative, high-value compounds. To illustrate the evolution and impact of the DBTL cycle in metabolic engineering, this review explores advancements in engineering *C. glutamicum*, focusing particularly on L-lysine-based chemical production. Beginning with the early development of L-lysine-producing strains, it examines how the expansion of the L-lysine biosynthesis pathway has enabled the production of a diverse range of C5 chemicals, including cadaverine, 5-aminovaleric acid (5-AVA), glutaric acid (GTA), 5-hydroxyvaleric acid (5-HV), 1,5-pentanediol (1,5-PDO), and valerolactam (VL). This review highlights the pivotal role of the DBTL cycle in establishing *C. glutamicum* as a robust and versatile platform for industrial biotechnology.

## From Traditional to Advanced Metabolic Engineering: Tools and Strategies for *C. glutamicum* Optimization Through the DBTL Cycle

### Traditional and systems metabolic engineering strategies for *C. glutamicum*

**Fig. 1.**
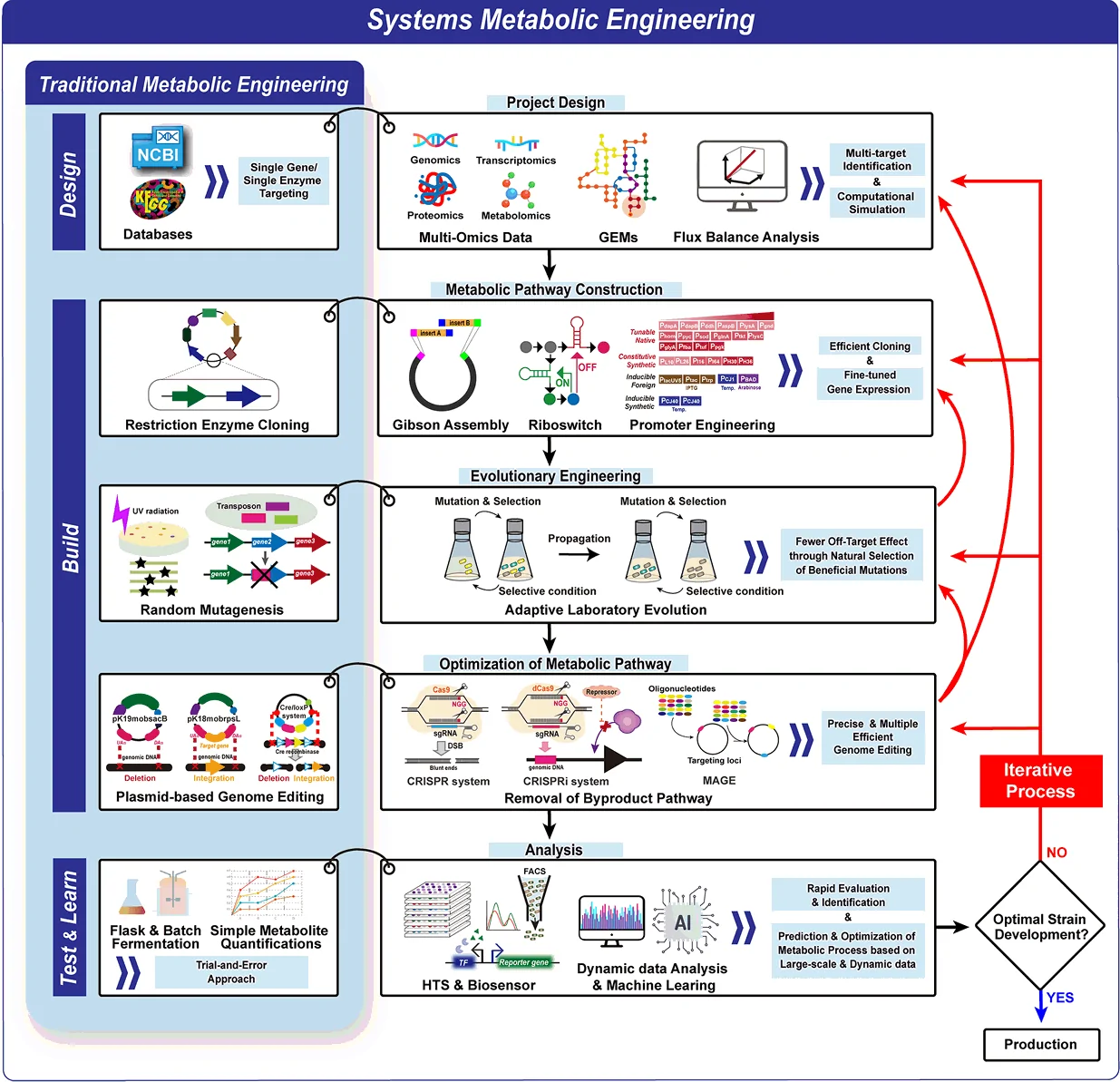
Transition from traditional to systems metabolic engineering via the Design-Build-Test-Learn framework. A comparison of traditional and advanced Design-Build-Test-Learn frameworks, emphasizing their iterative nature. Each step can be cycled through multiple times to enhance production efficiency or refined to improve methodologies and outcomes, enabling continuous improvement and optimization in metabolic engineering.

Traditional metabolic engineering strategies have primarily relied on trial-and-error-based gene-level modifications, typically involving pathway optimization through plasmid-based gene overexpression, chromosomal gene deletions, or the introduction of heterologous genes. During the design phase, pathway construction and reconstruction were limited to known metabolic pathways, available genome sequences, and basic metabolic flux analyses. In the build phase, gene knockouts and overexpression were commonly achieved using plasmid-based systems, while random mutagenesis approaches were also employed. These methods often required labor-intensive selection processes to identify *C. glutamicum* strains with desirable traits. As a result, the test phase was similarly constrained, relying primarily on phenotypic observations and simple metabolite quantification. Finally, in the learn phase, feedback loops were based on the manual interpretation of experimental data, which guided subsequent iterations of the engineering cycle (Fig. 1). Due to the inherent limitations of this rudimentary DBTL cycle, traditional metabolic engineering efforts primarily focused on the build phase, often targeting single pathways or straightforward metabolic objectives.

Systems metabolic engineering employs a more structured and iterative DBTL cycle, seamlessly integrating systems biology, synthetic biology, and evolutionary engineering. In the design phase, metabolic engineering projects are planned more comprehensively using advanced systems biology tools, such as genome-scale metabolic models (GEMs), multi-omics data analysis, and computational simulations. These approaches enable the identification of novel pathways and metabolic targets with greater precision. The build phase leverages state-of-the-art synthetic biology techniques for precise genome editing in *C. glutamicum*, including Clustered Regularly Interspaced Short Palindromic Repeats (CRISPR) and CRISPR-associated proteins (Cas) system, multiplex automated genome engineering (MAGE), and synthetic transposon systems. Additionally, pathway construction and expression are significantly enhanced through genetic circuits for dynamic regulation, modular pathway assembly, and multi-pathway integration, expanding the metabolic potential of the organism. In the test phase, strain evaluation goes beyond phenotypic observations to incorporate real-time assessments of cellular states. High-throughput screening (HTS) techniques and biosensors are employed to monitor strain performance, pathway efficiency, and metabolite production dynamically. Finally, in the learn phase, artificial intelligence (AI), machine learning (ML), and data-driven optimization refine predictive models and guide subsequent engineering cycles, ensuring continuous improvement. This iterative and data-driven DBTL framework facilitates rapid prototyping, predictive refinement, and the optimization of complex metabolic systems, enabling efficient exploration and exploitation of the metabolic networks in *C. glutamicum* (Fig. 1).

### Development of the DBTL cycle with a focus on the design phase

In traditional metabolic engineering, the design phase was largely confined to utilizing established databases such as Kyoto Encyclopedia of Genes and Genomes (KEGG) or National Center for Biotechnology Information (NCBI). These limitations restricted the scope of metabolic engineering projects to targeting individual genes or single enzymes. For example, in early efforts to engineer *C. glutamicum* for L-lysine production, aspartate kinase was a key target for manipulation, particularly for deregulation (see Section 3.1; Schrumpf et al., 1991).

With advancements in technology, the emergence of systems biology has significantly enhanced the design phase by integrating multi-dimensional biological data into comprehensive models of biological systems (Chuang et al., 2010). Innovations in computational tools and software have driven breakthroughs across various engineering disciplines, including biotechnology, enabling researchers to construct system-wide models, formulate new hypotheses, and analyze vast biological datasets to map and model cellular functions effectively. In metabolic engineering, tools such as GEMs, multi-omics data analysis—including transcriptomics, proteomics, and metabolomics—and computational simulations have become invaluable. GEMs facilitate the simulation of metabolic fluxes under diverse conditions, allowing for the identification of pathway bottlenecks and optimization targets. For example, while traditional design efforts were often limited to targeting a single gene or enzyme, such as aspartate kinase, a recent study introduced the iCW773 model for metabolic network reconstruction of *C. glutamicum* ATCC13032. This model was used to calculate flux variability ranges between wild-type and L-lysine-overproducing strains. Simulations identified key enzymes in the L-lysine synthetic pathway—including aspartate kinase, diaminopimelate dehydrogenase, dihydrodipicolinate synthase, diaminopimelate decarboxylase, and dihydrodipicolinate reductase—as critical regulatory points. These findings align with experimental reports, validating the effectiveness of the model in guiding metabolic engineering strategies (Zhang et al., 2017).

Additionally, multi-omics data analysis integrates diverse datasets to uncover regulatory mechanisms, gene expression patterns, and interactions among metabolites and enzymes. This comprehensive approach provides deeper insights, significantly improving strain design for industrial applications. For example, an integrated multi-omics analysis—including genome, transcriptome, and fluxome data—was applied to *C. glutamicum* BE, a strain that overproduces L-lysine. This analysis provided a detailed understanding of the mechanisms driving L-lysine overproduction and identified 11 target genes for pathway expansion toward GTA production. These included *zwf* (glucose-6-phosphate 1-dehydrogenase), the *tkt* operon, *ddh* (diaminopimelate dehydrogenase), *pgi* (glucose-6-phosphate isomerase), *icd* (isocitrate dehydrogenase), *lysE* (L-lysine exporter), *davB* (L-lysine monooxygenase from *Pseudomonas putida*), *davA* (5-aminovaleramide amidohydrolase from *P. putida*), *gabT* (4-aminobutyrate aminotransferase from *C. glutamicum*), *gabD* (succinate-semialdehyde dehydrogenase from *C. glutamicum*), and *ynfM* (GTA exporter). These findings offer a consolidated design framework, guiding metabolic manipulations through strategies such as promoter engineering, gene deletions, and the introduction of additional genes (Han et al., 2020). By incorporating advanced systems biology tools, the design phase in the DBTL cycle has evolved from a gene-centered approach to a comprehensive strategy capable of addressing complex metabolic networks. This transformation has enabled the development of highly efficient and tailored microbial cell factories for industrial applications.

### Development of the DBTL cycle with a focus on the build phase

In the early stages of metabolic engineering, the development of efficient microbial cell factories for *C. glutamicum* relied on genetic tools such as homologous recombination, traditional site-specific recombination, and random mutagenesis. These techniques were primarily based on natural or minimally modified molecular mechanisms. For example, homologous recombination was employed to introduce or delete genes using DNA templates with homologous arms. A widely used and fundamental genome editing tool for *C. glutamicum* is the *sacB* system, which facilitates homologous recombination-based genome modifications (Schäfer et al., 1994). As an allelic exchange tool, *sacB* has proven effective for various genomic modifications, including insertions, deletions, and substitutions (Kim et al., 2023). The *sacB* gene, derived from *Bacillus subtilis*, encodes the enzyme levansucrase, which is secreted into the culture medium, where it catalyzes sucrose hydrolysis and levan synthesis. Interestingly, the expression of *sacB* is lethal to *C. glutamicum* in the presence of sucrose. While this sensitivity is typically observed in gram-negative bacteria due to their periplasmic layer, it has been inferred that the structural composition of the *C. glutamicum* cell wall is responsible for the same phenomenon in this gram-positive species. These findings have established *sacB* as a powerful tool for insertional inactivation, facilitating the separation of transposable elements in *C. glutamicum* (Jäger et al., 1992; Tan et al., 2012). Consequently, the most commonly used recombination method in *C. glutamicum* involves the pK18mobsacB/pK19mobsacB plasmid system, which contains a kanamycin resistance gene-*sacB* cassette and enables efficient genetic manipulation (Schäfer et al., 1994). In addition to homologous recombination, site-specific recombinases have been employed for genome editing in *C. glutamicum*. One example is the Cre/*loxP*-mediated genome engineering system, which was developed as an advanced allelic exchange tool. Originally designed for genome rearrangements in eukaryotic cell lines, this system has also demonstrated effectiveness in promoting in vivo recombination in bacteria (Suzuki et al., 2005). The Cre/*loxP* mechanism, derived from bacteriophage P1, consists of two key components, i.e., Cre recombinase, which catalyzes site-specific recombination, and *loxP* sequences, which serve as recognition sites for this enzyme. The application of the Cre/*loxP* system begins with the strategic placement of *loxP* sites in identical orientations at both ends of the target genetic sequence via an integrated plasmid. Subsequently, a plasmid carrying the *cre* gene is introduced into the host cell, initiating recombination and leading to the excision of the intervening DNA sequence (Chai et al., 2021). The Cre/*loxP* recombination system offers a significant advantage over traditional homologous recombination due to its superior efficiency and versatility. Unlike conventional methods, it enables genomic modifications regardless of the length of the nucleotide sequence positioned between the two *loxP* sites, making it a highly valuable tool for genetic engineering in *C. glutamicum* (Suzuki et al., 2005).

Another strategy for genome editing in *C. glutamicum* involves applying random mutagenesis techniques. Chemical mutagens, such as nitrosoguanidine or ultraviolet (UV) radiation, introduce random mutations into the genome, followed by a selection process to isolate strains with desirable traits (Demain, 2000; Ikeda & Nakagawa, 2003; Shah et al., 2002). Alternatively, transposon mutagenesis provides a powerful approach for random mutagenesis in *C. glutamicum*. Transposons are mobile genetic elements capable of autonomously integrating into the genome, making them highly effective tools for genetic manipulation. The fully sequenced genome of *C. glutamicum* allows for precise transposon-based genetic modifications at both the gene and chromosome levels (Inui et al., 2005). Transposons are classified into distinct types, including Class I complex transposons, which consist of insertion sequence (IS) element pairs carrying additional genetic information—such as antibiotic resistance genes—and Class II transposons, which integrate features from more complex genetic elements like conjugative transposons, plasmids, and bacteriophages (Mormann et al., 2006; Suzuki et al., 2006). Several transposons have been identified and employed for genome engineering in *C. glutamicum* (Mormann et al., 2006), including IS31831, Tn31831 (Vertès et al., 1994), Tn14751 (Inui et al., 2005), Tn5 (Suzuki et al., 2006), ISCg2 (Quast et al., 1999), mini-Mu (Gorshkova et al., 2018), and IS1249 (Tauch et al., 1995). Among these, an artificial transposon, miniTn31831—derived from IS31831—was developed to facilitate random genomic insertions without requiring a specific target sequence, enhancing its utility as a genome engineering tool in *C. glutamicum* (Suzuki et al., 2006).

While traditional methods for constructing microbial cell factories present challenges in precisely mutating specific genes, innovative strategies have been developed to overcome these limitations. Synthetic biology aims to create synthetic variants of biological systems, allowing researchers to bypass inherent biological complexity and introduce novel functions or optimize existing processes (Esvelt & Wang, 2013). These rational and systematic approaches redefine biological systems, enabling the development of novel metabolic pathways and the engineering of more efficient strain variants. Key advancements in this field include cutting-edge genome editing techniques and the design of synthetic biological devices (Naseri & Koffas, 2020). Unlike conventional metabolic engineering tools, modern genome editing techniques offer superior precision, efficiency, scalability, and versatility.

For high-GC-content organisms such as *C. glutamicum*, the development of a highly specific CRISPR-Cas9 system holds significant potential for enhancing the accuracy of genomic modifications (Liu et al., 2017). The CRISPR-Cas9 system consists of two key components, i.e., CRISPR and Cas. Cas9, the most widely studied Cas protein, functions as an RNA-guided endonuclease that precisely cleaves target DNA sequences. The CRISPR RNA (crRNA) carries a guide sequence that directs Cas9 to its specific target site, enabling precise genomic alterations with broad applications in metabolic engineering and synthetic biology (Chen et al., 2017; Hsu et al., 2014; Huang et al., 2016; Paquet et al., 2016; Richardson et al., 2016; Shalem et al., 2014; Zerbini et al., 2017). Despite its transformative potential, Cas9 has been reported to exhibit toxicity in *C. glutamicum*, prompting the development of optimization strategies to mitigate these effects. One approach introduced an inducible P_tac_ promoter, allowing fine-tuned Cas9 expression through low concentrations of IPTG, effectively reducing toxicity (Peng et al., 2017). Additionally, an alternative genome regulation method, CRISPR interference (CRISPRi), has been successfully implemented in *C. glutamicum*. Unlike traditional CRISPR-Cas9 systems that cleave DNA, the catalytically inactive dCas9 forms a complex with single-guide RNA (sgRNA) and binds specifically to complementary DNA sequences. This binding effectively blocks the progression of RNA polymerase along the DNA strand, thereby inhibiting transcription. This mechanism is central to CRISPR interference (CRISPRi), which unlike genome-editing tools that create permanent mutations, offers reversible regulation of gene expression. By enabling the phenotype to be dynamically toggled 'ON' or 'OFF,' CRISPRi provides substantial flexibility, making it especially valuable in metabolic engineering where temporal control over gene expression can enhance production processes or study gene function more effectively (Cleto et al., 2016; Gilbert et al., 2013; Larson et al., 2013).

Evolutionary engineering strategies play a crucial role in the build phase of microbial cell factory development by harnessing natural selection to enhance strain performance. Adaptive laboratory evolution (ALE) is a particularly effective approach, allowing for the acquisition of complex phenotypes that are difficult to achieve through rational engineering alone. By leveraging natural selection, ALE optimizes target traits in production strains without requiring an in-depth understanding of underlying genetic mechanisms (Godara & Kao, 2020; Prell et al., 2021; Sandberg et al., 2019). This process is typically conducted through iterative culturing under controlled conditions, including exposure to selective pressures such as pH adjuvants, lethal substrates, or mutagenic agents to induce beneficial mutations (Dragosits & Mattanovich, 2013; Mans et al., 2018; Stella et al., 2019). Additional factors, such as nutritional limitations and UV irradiation, can also be employed to accelerate adaptive changes (González-Ramos et al., 2016; Wang et al., 2023). By subjecting microbial populations to these specific environmental pressures, ALE harnesses natural selection to overcome constraints associated with limited knowledge of intricate metabolic and cellular processes (Stella et al., 2019). The resulting pool of evolved strains can then be rapidly analyzed using HTS and biosensor-assisted selection, enabling efficient identification of strains with enhanced performance characteristics.

### Development of the DBTL cycle with a focus on the test phase

The test phase of the DBTL cycle has traditionally relied on flask and batch fermentations, followed by analytical techniques such as high-performance liquid chromatography and enzyme assays. However, advancements in synthetic biology and evolutionary engineering have significantly improved this phase by enabling the diversification and optimization of engineered strains through phenotype-based selection. These innovations, combined with HTS and biosensor-assisted methods, have streamlined strain evaluation, increasing efficiency and precision. In *C. glutamicum*, a broad-host-range synthetic small RNA (BHR-sRNA) system has been developed by integrating the *B. subtilis* RoxS scaffold with the Hfq chaperone. When paired with HTS techniques such as colorimetric assays, this system enables the rapid knockdown of up to 2,959 genes within just 2–3 days, demonstrating its potential for accelerating strain testing and optimization (Cho et al., 2023). Similarly, the integration of evolutionary engineering with biosensors has introduced groundbreaking solutions to overcome limitations in engineered strains. For example, Stella et al. (2021) developed a biosensor-driven synthetic circuit in *C. glutamicum* to enhance small molecule production. By placing the Lrp biosensor upstream of key metabolic genes such as *pfkA* and *hisD*, the circuit linked intracellular branched-chain amino acid concentrations to strain growth. This approach enabled the selection of mutant strains with amino acid production, illustrating an innovative method for addressing metabolic bottlenecks.

Furthermore, biosensor-assisted evolutionary engineering offers a powerful framework for investigating mutations identified during the build phase, enabling targeted mutagenesis for strain improvement. Binder et al. (2013) exemplified this approach by developing a rapid recombination and screening system that utilized the RecT recombinase from prophage Rac in combination with the pSenLys sensor. This system successfully identified mutations in *murE* that influenced L-lysine production, demonstrating its effectiveness in accelerating strain optimization. These advancements highlight the transformative impact of biosensors and synthetic biology tools on the test phase, providing modern solutions for more efficient strain evaluation and accelerating the development of microbial cell factories.

### Development of the DBTL cycle with a focus on the learn phase

In traditional learn-phase approaches, researchers have often relied on trial-and-error methods due to the limited insights available from the test phase. Early studies on L-lysine production in *C. glutamicum* primarily examined mutations within the L-lysine biosynthesis pathway, overlooking secondary mutations and their combinatorial effects. This approach, constrained by reliance on previously established systems, provided only a fragmented understanding of metabolic dynamics and lacked predictive capabilities. In contrast, systems biology enables the modeling of metabolic processes and signaling networks at the cellular level, allowing for the prediction and optimization of metabolic behaviors. Advances in data-processing technologies now empower researchers to analyze large-scale biological data streams, generate hypotheses, and iteratively test them. For instance, L-lysine production in *C. glutamicum* imposes significant metabolic demands, particularly for NADPH, oxaloacetate, and pyruvate. These limitations can be effectively addressed through metabolic flux analysis (MFA). The value of MFA has been demonstrated using 13C tracer studies, isotope modeling, and gas chromatography-mass spectrometry to analyze *C. glutamicum* metabolic networks during L-lysine production from glucose or fructose (Kiefer et al., 2004). Findings indicate that the flux through the pentose phosphate pathway (PPP) is significantly reduced when fructose is used instead of glucose, while pyruvate dehydrogenase flux increases by approximately 25%. Additionally, the constrained NADPH supply from fructose was identified as a key bottleneck in L-lysine production, which could be alleviated by enhancing malic enzyme activity. These insights into substrate utilization, enabled by systems biology, provide a strong foundation for defining engineering targets such as key enzymes or promoters (Kiefer et al., 2004).

## Broadening the Metabolic Potential of *C. glutamicum*: From L-lysine production to Specialty Chemical Biosynthesis

### Advancing *C. glutamicum* for L-lysine overproduction

**Fig. 2.**
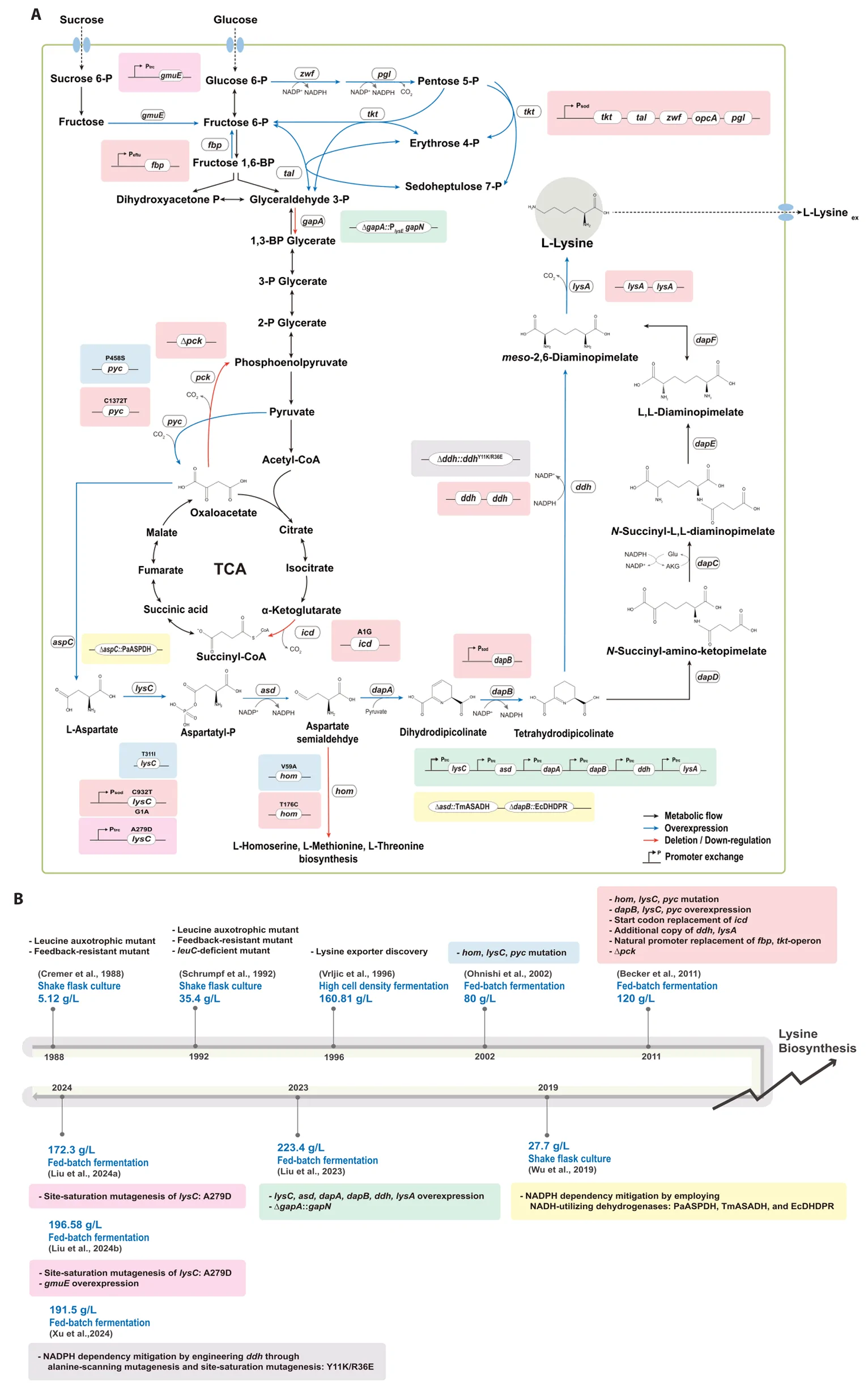
(A) Metabolic pathway for L-lysine production in *C. glutamicum*. A schematic representation of the L-lysine biosynthetic pathway in *C. glutamicum*, incorporating genetic modifications discussed in the text to enhance L-lysine overproduction. Gene abbreviations: glucose-6-phosphate 1-dehydrogenase (*zwf*), transketolase (*tkt*), glucose-6-phosphate isomerase (*pgi*), isocitrate dehydrogenase (*icd*), transaldolase (*tal*), PEP carboxykinase (*pck*), pyruvate carboxylase (*pyc*), aspartate semialdehyde dehydrogenase (*asd*), dihydrodipicolinate synthase (*dapA*), dihydrodipicolinate reductase (*dapB*), homoserine dehydrogenase (*hom*), diaminopimelate dehydrogenase (*ddh*), N-succinyl-amino-ketopimelate transaminase (*dapC*), tetrahydrodipicolinate succinylase (*dapD*), N-succinyl-diaminopimelate desuccinylase (*dapE*), diaminopimelate epimerase (*dapF*), diaminopimelate decarboxylase (*lysA*), *NADH-utilizing dehydrogenases* (*PaASPDH*, *TmASADH*, and *EcDHDPR*), L-lysine exporter (*lysE*), putative fructokinase (*gmuE*), fructose-1,6-bisphosphatase (*fbp*), outer membrane adhesin/invasin (*opcA*), gamma-aminobutyric acid receptor (*gabA*), NADP-dependent glyceraldehyde-3-phosphate dehydrogenase (*gabN*), and aspartate aminotransferase (*aspC*). (B) Evolution of L-lysine production in *C. glutamicum*. A timeline illustrating advancements in L-lysine production, transitioning from traditional metabolic engineering to systems metabolic engineering. Each colored box corresponds to the genomic modifications highlighted in (A): pink (Liu et al., 2024a, 2024b), purple (Xu et al., 2024), green (Liu et al., 2023), yellow (Wu et al., 2019), orange (Becker et al., 2011), and blue (Ohnishi et al., 2002).

The history of L-lysine production using *C. glutamicum* illustrates the progression of industrial biotechnology, from traditional microbial fermentation to advanced systems metabolic engineering. L-lysine, an essential amino acid with significant applications in animal feed, human nutrition, and pharmaceuticals, was among the first amino acids to be industrially produced using microorganisms (Cheng et al., 2018; Eggeling & Bott, 2015; Felix et al., 2019; Liu et al., 2022). L-lysine is synthesized via the aspartate pathway in prokaryotes, plants, and some fungi. *C. glutamicum* is particularly well-suited for industrial L-lysine production due to its highly efficient biosynthetic capabilities. The pathway comprises multiple enzymatic steps, beginning with aspartate and culminating in L-lysine synthesis (Fig. 2).

L-lysine biosynthesis begins with the conversion of oxaloacetate (OAA), a key intermediate in the TCA cycle, into aspartate via aspartate aminotransferase. Aspartate is then phosphorylated by aspartate kinase (LysC) to form aspartylphosphate, a step tightly regulated through feedback inhibition by L-lysine and methionine. Aspartate semialdehyde dehydrogenase (Asd) subsequently converts aspartylphosphate into aspartate semialdehyde, which serves as a crucial branching point for the biosynthesis of L-lysine, methionine, and threonine. Dihydrodipicolinate synthase (DapA) catalyzes the condensation of aspartate semialdehyde and pyruvate, leading to the formation of dihydrodipicolinate. This intermediate is then reduced by dihydrodipicolinate reductase (DapB) to generate tetrahydrodipicolinate. From this point, *meso*-2,6-diaminopimelate synthesis follows one of two distinct pathways, depending on the enzymatic system employed. The multi-step pathway involves a sequential conversion of tetrahydrodipicolinate via four key enzymes: tetrahydrodipicolinate succinylase (DapD), N-succinyl-amino-ketopimelate transaminase (DapC), N-succinyl-diaminopimelate desuccinylase (DapE), and diaminopimelate epimerase (DapF). Initially, DapD transfers a succinyl group from succinyl-CoA to tetrahydrodipicolinate, forming N-succinyl-amino-ketopimelate. DapC then introduces an amino group through transamination, followed by DapE-mediated hydrolysis to remove the succinyl group, yielding L,L-diaminopimelate. This intermediate is subsequently rearranged by DapF to produce *meso*-2,6-diaminopimelate. Alternatively, a single-step pathway bypasses these intermediate reactions through diaminopimelate dehydrogenase (Ddh), which directly converts tetrahydrodipicolinate into *meso*-2,6-diaminopimelate via reductive amination, utilizing ammonium and NADPH as cofactors. The Ddh pathway provides a more direct and efficient route, circumventing the need for succinylation and hydrolysis. Regardless of the pathway taken, *meso*-2,6-diaminopimelate is decarboxylated by diaminopimelate decarboxylase (LysA) to yield L-lysine. The presence of both pathways highlights the metabolic flexibility of *C. glutamicum*, allowing it to adapt L-lysine biosynthesis based on environmental and cellular conditions (Eikmanns et al., 1993). Among them, the Ddh-based pathway has been shown to support a higher metabolic flux capacity compared to the multi-step DapDCEF pathway, making it a more favorable route for L-lysine production (Schrumpf et al., 1991).

The discovery of *C. glutamicum* laid the foundation for large-scale microbial amino acid production, demonstrating its potential for the overproduction of a diverse range of amino acids (Cremer et al., 1988; Luntz et al., 1986; Schrumpf et al., 1991). Early efforts to develop L-lysine-producing strains relied on classical mutagenesis techniques, including chemical mutagens such as nitrosoguanidine and UV irradiation. These approaches facilitated the generation of auxotrophic mutants with deregulated biosynthetic pathways, effectively enhancing L-lysine production by circumventing feedback inhibition and regulatory constraints (Eikmanns et al., 1993; Plachý et al., 1985). A key strategy involved mutagenizing *C. glutamicum* wild-type strains and screening colonies on minimal media supplemented with L-lysine analogs such as L-threonine and D,L-S-2-aminoethyl-L-cysteine. These analogs mimic the intracellular L-lysine environment and inhibit aspartate kinase, the first committed step in L-lysine biosynthesis. Mutants exhibiting resistance to this inhibition were selected at a frequency of approximately 1.3 × 10^-3^ after mutagenesis, compared to 2.2 × 10^-5^ in untreated cells. Among 96 screened mutants, seven were found to secrete L-lysine, with strain MH20 accumulating up to 35 mM L-lysine in flask cultures (Cremer et al., 1988). The regulation and activity of L-lysine biosynthetic enzymes were further analyzed under batch culture conditions, revealing that L-lysine production is influenced by various enzymatic activities. Diaminopimelate decarboxylase, the enzyme responsible for the final step of L-lysine biosynthesis, undergoes a significant reduction in activity in the presence of L-lysine, decreasing to approximately one-third of its original level. However, other key enzymes in the pathway, such as aspartate-semialdehyde dehydrogenase and dihydrodipicolinate synthase, remain unaffected by amino acids from the aspartate family. L-homoserine dehydrogenase (Hom) exhibits a distinct regulatory response to different amino acids. In the presence of L-methionine, its activity is repressed to 15% of its original level, while exposure to L-threonine leads to an even stronger inhibition, reducing activity to just 4%. Conversely, L-leucine exerts a stimulatory effect, doubling Hom activity. This redirection of aspartate semialdehyde flux toward homoserine consequently diminishes L-lysine biosynthesis, highlighting the potential of L-leucine auxotrophic mutants as a strategy for improving L-lysine overproduction. Further optimization of L-lysine production has been demonstrated through targeted genetic modifications. For example, overexpressing dihydrodipicolinate synthase by introducing the *Escherichia coli* *dapA* gene significantly increased metabolic flow toward L-lysine, underscoring the potential of rational genetic engineering to enhance L-lysine biosynthesis in *C. glutamicum* (Cremer et al., 1988).

Further advancements in L-lysine production were achieved through the introduction of auxotropic mutations, notably L-leucine auxotrophy, which significantly increased L-lysine yields. This improvement was attributed to a deficiency in isopropylmalate dehydratase activity, effectively redirecting metabolic flux from L-leucine biosynthesis toward L-lysine production (Schrumpf et al., 1992; Tosaka et al., 1978). Building on this strategy, the derivative strain MH20-22B was developed from MH20 using a two-step mutagenesis process. When cultivated in a simple mineral salt medium with 100 g/L glucose, MH20-22B achieved an impressive L-lysine HCl concentration of 44 g/L. The strain also exhibited a high L-lysine secretion rate of 0.57 mmol/g dry weight per h, a substantial increase compared to 0.19 mmol/g per h in MH20. Enzymatic analysis revealed that MH20-22B possessed a feedback-resistant aspartate kinase while lacking isopropylmalate dehydratase activity, which contributed to enhanced L-lysine biosynthesis. Interestingly, despite the significant increase in L-lysine secretion, cytosolic L-lysine concentrations in MH20 and MH20-22B remained comparable. This observation suggested that unknown mutations, likely within the L-lysine export system, played a crucial role in enhancing secretion efficiency (Schrumpf et al., 1992). Further investigation led to the identification of *lysE* as the key gene responsible for L-lysine excretion. A *lysE*-deficient mutant exhibited a complete loss of L-lysine secretion, whereas overexpression of *lysE* significantly improved the L-lysine excretion rate, ultimately enabling the production of up to 1,100 mM L-lysine (Vrljic et al., 1996).

### Enhancing *C. glutamicum* for L-lysine hyperproduction through systems metabolic engineering

Building on insights from traditional random mutagenesis strategies—which often introduce uncharacterized secondary mutations that may impair strain performance—the post-genomic era introduced genome-based strain reconstruction as a precise alternative (Ohnishi et al., 2002). This approach enables the identification of beneficial mutations through comparative genomic analysis and their systematic reintroduction into a wild-type background, allowing for targeted strain improvement. For example, comparative genomic analysis between a wild-type strain and a high-producing mutant identified key mutations in pyruvate carboxylase (*pyc*; P458S), *hom* (V59A), and *lysC* (T311I). By sequentially incorporating these mutations into the wild-type genome, researchers developed *C. glutamicum* AHP-3, a strain that exhibited a synergistic enhancement in L-lysine production. During fed-batch fermentation, AHP-3 achieved a final titer of 80 g/L and a record productivity of 3.0 g/L/h (Ohnishi et al., 2002). Building on this comparative genomic approach, subsequent studies expanded their focus to include upstream pathways, particularly the PPP, as well as downstream pathways of L-lysine biosynthesis. These studies identified 6-phosphogluconate dehydrogenase (Gnd) as a key metabolic target, with the S361F mutation playing a pivotal role in optimizing L-lysine production. Introducing this mutation into *C. glutamicum* AHP-3 led to a 1.15-fold increase in L-lysine yield. Metabolic flux analysis using isotope labeling further revealed that the *gnd* mutation enhanced carbon flux through the PPP by 8%, resulting in increased availability of NADPH—a crucial cofactor in L-lysine biosynthesis. The enhanced NADPH supply further optimized the metabolic process, demonstrating the power of targeted genetic modifications in strain engineering (Ohnishi et al., 2005).

A comprehensive multi-omics analysis of *C. glutamicum* ATCC 13287 during batch fermentation highlighted the critical role of key enzymes in glycolysis, the PPP, the TCA cycle, and L-lysine biosynthesis in regulating transcription (Kromer et al., 2004). By integrating ^13^C flux analysis with isotopomer modeling, DNA microarray-based expression profiling, and intracellular metabolite quantification, significant metabolic and regulatory shifts were revealed. The transition from growth to L-lysine production was marked by a decrease in glucose uptake flux, a redirection of carbon flux from the TCA cycle to anaplerotic reactions and L-lysine biosynthesis, and dynamic changes in intracellular metabolite pools, including the accumulation of L-lysine up to 40 mM before excretion. Correlations between gene expression and metabolic flux were observed for key TCA cycle enzymes such as transaldolase and glucose-6-phosphate dehydrogenase (G6PD). Interestingly, malate dehydrogenase exhibited increased expression despite reduced TCA flux, likely due to its role in NADH regeneration, emphasizing the complex interplay between regulatory networks and metabolic flux (Kromer et al., 2004). Leveraging insights from systems biology, L-lysine hyperproducing strains have been developed through advanced systems metabolic engineering strategies. One notable example involved in silico modeling, which identified 12 genome-based modifications to optimize L-lysine production. These modifications involved nucleotide exchanges and the overexpression of feedback-resistant *lysC*, along with the introduction of additional copies of *ddh* and *lysA*. To enhance OAA supply, *pck* was deleted while *pyc* was overexpressed. Further adjustments included the overexpression of *dapB* and the introduction of a reduced-activity mutation in *hom* (V59A). Moreover, the start codon in *icd* was replaced to redirect TCA cycle flux toward L-lysine, while the *tkt* operon was overexpressed to increase PPP flux. Through these targeted genetic modifications, the central metabolic network of *C. glutamicum* was systematically restructured to redirect major carbon fluxes toward L-lysine biosynthesis. The final engineered strain demonstrated impressive production metrics, achieving a productivity of 4.0 g/L/h, an L-lysine titer of 120 g/L, and a yield of 0.55 g/g glucose in fed-batch cultures (Becker et al., 2011). In another study, feedback inhibition was rationally deregulated to enhance OAA, a key L-lysine precursor, and its impact on metabolic flux redistribution was investigated. Guided by computational modeling, a point mutation (N917G) in the *ppc* gene was introduced to enhance phosphoenolpyruvate carboxylase (PEPC) activity, thereby improving OAA availability. This resulting strain yielded 0.211 mol L-lysine/mol glucose by efficiently controlling central metabolic fluxes (Chen et al., 2014). More recently, sequence alignment and functional analysis of aspartate kinases from different L-lysine-producing strains revealed that the 279th residue plays a pivotal role in reducing inhibition by L-threonine and L-lysine. Site-saturation mutagenesis of this residue identified *lysC*(A279D)—where alanine was replaced by aspartate—as the most effective variant in minimizing inhibitor interactions. The engineered strain LYS-279D, overexpressing *lysC*(A279D), achieved enhanced L-lysine production, reaching a titer of 172.3 g/L, a productivity of 2.39 g/(L·h), and a glucose conversion efficiency of 47.74% during fed-batch fermentation (Liu et al., 2024b).

The biosynthesis of L-lysine is highly dependent on NADPH, requiring 4 mol of NADPH for the synthesis of 1 mol of L-lysine. This reliance suggests that enhancing NADPH regeneration could significantly improve production efficiency. In *C. glutamicum*, the PpnK catalyzes the phosphorylation of NAD/NADH to produce NADP^+^. Functioning as a homotetramer, PPNK exhibits higher catalytic efficiency with ATP than with PolyP, and its activity is influenced by bivalent cations, particularly magnesium and manganese. Overexpression of *ppnK* in an L-lysine-producing strain increased yield by 12%, achieving 0.136 mol L-lysine per mol of glucose (Lindner et al., 2010). To address the NADPH dependency of L-lysine biosynthesis, another study employed enzyme mining to identify NADH-utilizing dehydrogenases. This approach uncovered several candidates, including aspartate aminotransferase from *Pseudomonas aeruginosa* (PaASPDH), Asd from *Tistrella mobilis* (TmASADH), DapB from *E. coli* (EcDHDPR), and Ddh from *Pseudothermotoga thermarum* (PtDAPDH). Overexpression of these enzymes individually enhanced L-lysine production by up to 36.8% in a basic L-lysine producer strain. In an L-lysine hyperproducer strain, combinatorial replacement of NADPH-dependent enzymes further improved production, with the highest increase (30.7%) observed in a triple-mutant strain co-expressing PaASPDH, TmASADH, and EcDHDPR, which achieved an L-lysine titer of 27.7 g/L (0.35 g/g glucose). A quadruple-mutant strain incorporating all four enzymes yielded 24.1 g/L (0.30 g/g glucose) while reducing reliance on the oxidative PPP (Wu et al., 2019). Further efforts to shift cofactor preference from NADPH to NADH in *C. glutamicum* focused on engineering Ddh through alanine-scanning mutagenesis. This analysis identified Tyr11, Arg36, and Arg37 as critical residues influencing cofactor specificity. Subsequent site-saturation mutagenesis and combinatorial analysis yielded the Ddh(Y11K/R36E) variant, which exhibited enhanced NADH utilization. Overexpression of this mutant in the *C. glutamicum* XQ-7 strain significantly enhanced L-lysine production, achieving a titer of 191.5 g/L with a yield of 0.53 g/g glucose (Xu et al., 2024). In addition to enzyme engineering, dynamic regulation of NADPH availability was explored using a promoter library responsive to intracellular L-lysine levels. By modifying the L-lysine-sensitive LysG regulator and the p*lysE* promoter, researchers developed a synthetic promoter library with varying strengths to control gene expression. These promoters were applied to regulate NADPH regeneration genes, such as *gapN*, in an L-lysine-responsive manner. Dynamic *gapN* regulation significantly improved NADPH availability, supporting efficient L-lysine biosynthesis while maintaining redox homeostasis. However, excessive NADPH accumulation disrupted metabolic balance, emphasizing the need for finely tuned promoter activity. The optimized strain, LYS-6, demonstrated an impressive 75% improvement in L-lysine production, achieving a titer of 80.9 g/L. Further fed-batch fermentation in a 5 L bioreactor yielded 223.4 g/L of L-lysine, highlighting the industrial potential of the strain (Liu et al., 2023).

These cases demonstrate how rational systems metabolic engineering, driven by advancements in systems biology, synthetic biology, and evolutionary engineering, has evolved into a comprehensive strategy for optimizing L-lysine production. By combining targeted genetic modifications with precise metabolic flux regulation, these approaches highlight the potential of systematic strain design in industrial biotechnology.

### Leveraging L-lysine hyperproducing *C. glutamicum* for specialty chemical synthesis

**Fig. 3.**
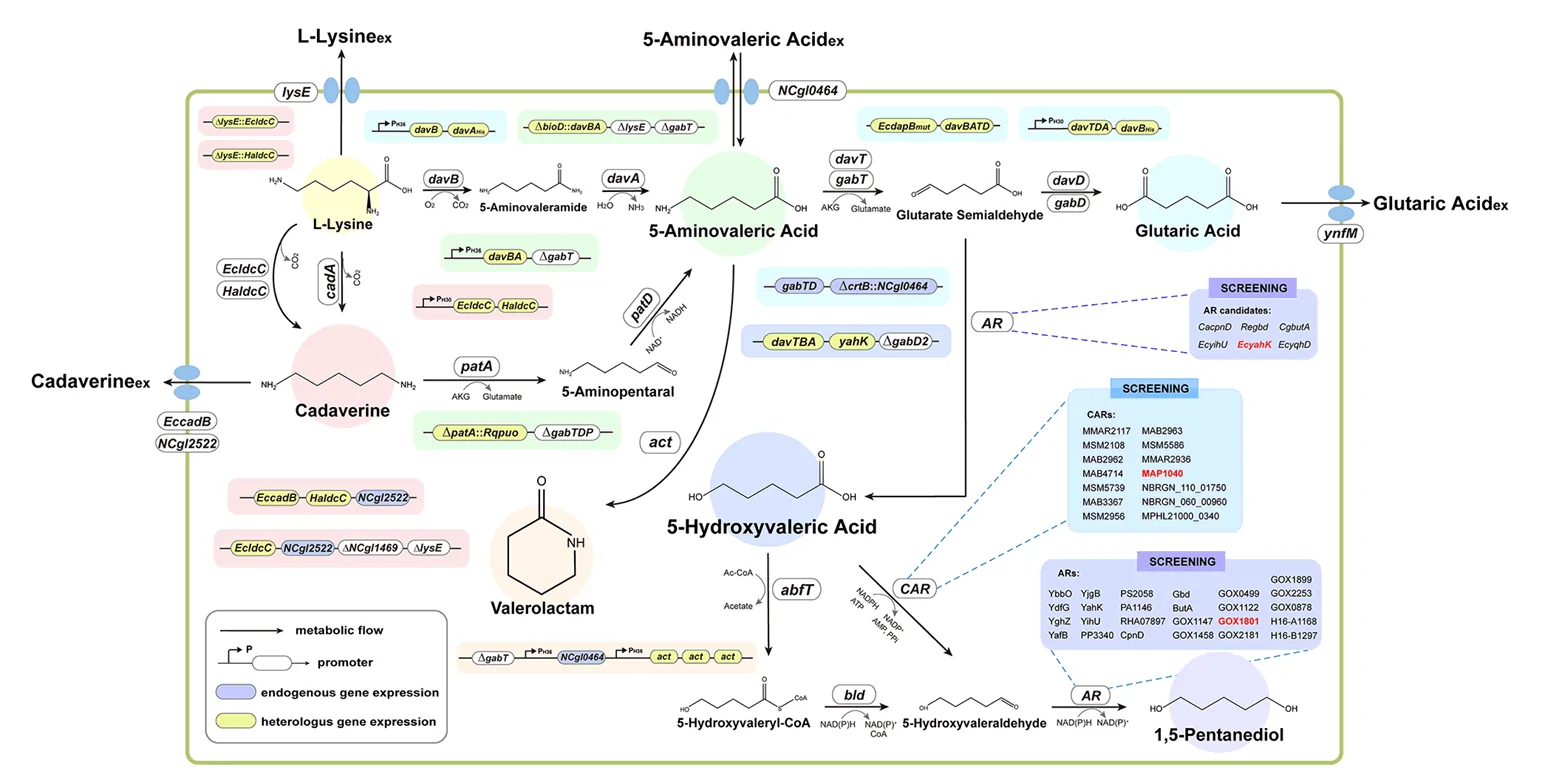
Expansion of the L-lysine biosynthesis pathway into C5 specialty chemicals via systems metabolic engineering in *C. glutamicum*. A schematic representation of the metabolic engineering strategies used to convert L-lysine into various C5 specialty chemicals, as described in the text. The metabolic pathway modifications for each product are color-coded: cadaverine (pink), 5-aminovaleric acid (green), 5-hydroxyvaleric acid (blue), and valerolactam (orange). Introduce foreign genes that enable pathway construction are indicated along metabolic flow arrows, with key genes selected through screening highlighted in red. Gene abbreviations: L-lysine exporter (*lysE*), L-lysine decarboxylase (*cadA*), L-lysine-cadaverine antiporter (*EccadB*), constitutive lysine decarboxylase from *H. alvei* (*HaldcC*), constitutive L-lysine decarboxylase from *E. coli* (*EcldcC*), cadaverine exporter (*NCgl2522*), N-acetyltransferase (*NCgl1469*), putrescine oxidase (*puo*), putrescine transaminase (*patA*), aminobutyraldehyde dehydrogenase (*patD*), L-lysine monooxygenase (*davB*), 5-aminovaleramide amidohydrolase (*davA*), 5-aminovaleric acid aminotransferase (*davT*), β-alanine CoA transferase (*act*), 4-aminobutyrate transporter (*PP2911*), amino acid permease (*NCgl0464*), succinate-semialdehyde dehydrogenase (*gabD*), 4-aminobutyrate aminotransferase (*gabT*), glutarate exporter (*ynfM*), carboxylic acid reductase (*CAR*), 4-hydroxyburtyl-CoA-transferase (*abfT*), butyraldehyde dehydrogenase (*bld*), aldehyde reductase (*EcyahK*), carboxypeptidase N deficiency (*CacpnD*), multiple reductase (*Regbd*), diacetyl reductase (*CgbutA*), 3-sulfolactaldehyde (*EcyihU*), and additional aldehyde reductases (*EcyahK*, *EcyqhD*).

Expanding on the development of L-lysine hyperproducing *C. glutamicum*, the biosynthetic pathway has been strategically engineered to facilitate the production of a diverse range of value-added chemicals. Through the incorporation of additional metabolic circuits, L-lysine flux has been redirected toward the synthesis of various C5 compounds, including cadaverine, 5-AVA, and GTA (Table 1, Fig. 3). These advancements highlight the adaptability of L-lysine hyperproducing strains as versatile platforms for specialty chemical production.

Cadaverine production is primarily driven by the decarboxylation of L-lysine, a reaction catalyzed by L-lysine decarboxylase (LDC) (Kind et al., 2010). Since *C. glutamicum* lacks the natural ability to produce cadaverine, an initial engineering strategy introduced the inducible *E. coli* *cadA* gene into the *hom* locus, generating an L-homoserine auxotroph that produced 2.6 g/L of cadaverine after 18 h of cultivation (Mimitska et al., 2007). Further advancements were achieved by co-expressing *cadB*, a cadaverine-L-lysine antiporter from *E. coli*, alongside *ldcC* from *Hafnia alvei*, increasing cadaverine titers to 2.75 g/L. Additional overexpression of *NCgl2522*, a cadaverine exporter, further improved yield by 20% (Li et al., 2014). A more refined approach involved modifying *C. glutamicum* LYS-12 by introducing a codon-optimized version of *E. coli* *ldcC*, overexpressing *NCgl2522* (permease), and deleting *NCgl1469* (N-acetyltransferase) and *lysE*. The resulting strain, *C. glutamicum* DAP-16, achieved a molar conversion efficiency of 50% cadaverine per mol of glucose, with a final titer of 88 g/L and a productivity of 2.2 g/L/h during fed-batch fermentation (Kind et al., 2014). In a separate study, *C. glutamicum* KCTC 1857 expressing *E. coli* *ldcC* under the strong H30 promoter reached a production level of 40.9 g/L of cadaverine (Oh et al., 2015). More recently, *C. glutamicum* PKC was engineered by integrating *E. coli* *ldcC* or *H. alvei* *ldcC* into the *lysE* locus, resulting in strains *C. glutamicum* G-H30 and GH30HaLDC, which achieved cadaverine titers of 104 g/L and 125 g/L, respectively (Kim et al., 2018, 2019a).

5-AVA is a key intermediate in the L-lysine degradation pathway, facilitating its conversion to GTA via the aminovaleric acid pathway, with 5-AVA and glutarate semialdehyde as key intermediates. In *C. glutamicum*, two distinct biosynthetic routes for 5-AVA production have been established: one utilizing L-lysine monooxygenase (*davB*) and 5-aminovaleramidase (*davA*) from *P. putida*, and another relying on L-lysine decarboxylase (*ldcC*) from *E. coli*, along with putrescine transaminase (*patA*) and aminobutyraldehyde dehydrogenase (*patD*), which converts cadaverine into 5-aminopentanal before its transformation into 5-AVA. The *davBA*-mediated pathway was first demonstrated for 5-AVA synthesis in *E. coli* (Adkins et al., 2013; Park et al., 2013) and later optimized in *C. glutamicum* through targeted metabolic engineering. For example, the L-lysine-overproducing strain *C. glutamicum* LYS-12 (Becker et al., 2011) was engineered by integrating *davBA* into the *bioD* locus while deleting *lysE* and *gabT*, leading to a production level of 28 g/L during fed-batch fermentation (Rohles et al., 2016). Further refinements using the expired commercial strain *C. glutamicum* BE included transcriptional enhancement of codon-optimized *davBA* under the robust synthetic PH36 promoter, achieving 19.7 g/L of 5-AVA. Additional deletion of *gabT*, which redirected flux away from GTA formation, increased production to 33.1 g/L (Shin et al., 2016). Leveraging systems metabolic engineering, production was further enhanced to 46.5 g/L with a selectivity of 0.34 g/g glucose by incorporating *P. putida* *davBA* while systematically eliminating competing pathways responsible for by-product formation. This highlighted the efficacy of integrating synthetic biology and systems-level approaches in optimizing 5-AVA biosynthesis. Notably, deletion of the GTA production pathway, alongside optimization of 5-AVA export and import systems, significantly improved cell growth and 5-AVA production efficiency (Rohles et al., 2022). An alternative route, the *ldcC*-*patA*D pathway, converts L-lysine to cadaverine, which is subsequently transformed into 5-AVA. Eliminating by-products such as N-acetylcadaverine, cadaverine, and GTA improved 5-AVA accumulation, reaching a titer of 5.1 g/L from 39.23 g/L of glucose. This pathway also exhibited flexibility in utilizing alternative feedstocks, including starch, glucosamine, and xylose, yielding comparable production levels to glucose and positioning glucosamine as a particularly promising feedstock (Jorge et al., 2017). Additionally, replacing *patA* with putrescine oxidase (*puo*) from *R. qingshengii*, in combination with *gabTDP* deletion, led to a production of 3.7 g/L of 5-AVA (Haupka et al., 2020). These advancements underscore significant progress in engineering *C. glutamicum* for efficient 5-AVA production.

With its ability to extend the 5-AVA biosynthesis pathway toward GTA production, *C. glutamicum* has emerged as a promising microbial host for GTA synthesis. The native *gabTD* genes in *C. glutamicum* catalyze the conversion of 5-AVA to GTA, efficiently utilizing the abundant precursors supplied by the *davBA*-mediated pathway. In fed-batch cultivation, a *C. glutamicum* strain expressing *davB* alongside codon-optimized, His-tagged *davA* under the control of the robust synthetic P_H36_ promoter produced 19.7 g/L of 5-AVA, accompanied by a substantial by-product accumulation of 13.4 g/L GTA (Shin et al., 2016). To enhance GTA production, heterologous expression of *davBATD* from *P. putida* was introduced. A recombinant *C. glutamicum* KCTC 1857 strain, engineered to express codon-optimized *davTDA* and His-tagged *davB* under the strong synthetic P_H30_ promoter, achieved GTA yields of 24.5 g/L, alongside 1.7 g/L of residual L-lysine during fed-batch fermentation (Kim et al., 2019b). Further optimization was accomplished through the development of a double-vector system incorporating *davBATD* alongside a mutant *E. coli* *dapB_mut_* (*dapB^C115G,G116C^*), which played a crucial role in regulating NADH availability—a key cofactor in the L-lysine biosynthesis pathway. This engineered biosynthetic system enabled a significant increase in GTA production, reaching approximately 65.6 g/L, with complete conversion of intermediates (Sohn et al., 2022). Additional improvements involved overexpressing the native *gabTD* operon and integrating the amino acid permease *NCgl0464* into the *crtB* locus, further boosting GTA production to over 90 g/L from glucose (Rohles et al., 2018). Recent advancements in systems metabolic engineering have continued to revolutionize GTA biosynthesis in L-lysine-producing *C. glutamicum*. By designing and optimizing synthetic pathways through genome-scale metabolic simulations, flux response analysis, and comparative transcriptome analysis, researchers have further refined production efficiency. Notably, the incorporation of *ynfM* and the implementation of a pH-stat feeding strategy during fed-batch cultivation resulted in a remarkable GTA yield of 105.3 g/L, with no detectable by-product formation (Han et al., 2020).

### Systems metabolic engineering for expanding L-lysine pathways into specialty chemical synthesis

While section 3.3 covered the initial expansion of the L-lysine biosynthesis pathway, primarily from the late 2000s to the 2010s, this section highlights more recent advancements driven by systems metabolic engineering. These developments have enabled the efficient production of C5 specialty chemicals, including 5-HV, 1,5-PDO, and VL (Fig. 3, Table 1).

Alongside GTA production, the biosynthesis of 5-HV has been enabled by extending the 5-AVA pathway, leading to the development of *C. glutamicum* strains optimized for high-level 5-HV production. A synthetic pathway was constructed by incorporating key steps from the L-lysine catabolic pathway, beginning with the conversion of L-lysine to 5-aminovaleramide, followed by an intracellular reduction step. Specifically, the *P. putida* *davTBA*-encoded pathway catalyzed the transformation of L-lysine into glutaric acid semialdehyde, which was subsequently reduced to 5-HV using an aldehyde reductase. To identify the most effective reductase, multiple candidates—including Gbd from *Ralstonia eutropha*, ButA from *C. glutamicum*, CpnD from *Clostridium aminovalericum*, and YahK, YqhD, and YihU from *E. coli*—were evaluated, with YahK emerging as the most efficient enzyme for 5-HV production. To minimize GTA by-product formation, the endogenous *gabD2* gene, responsible for glutaric acid semialdehyde oxidation, was deleted. In fed-batch fermentation, the engineered *C. glutamicum* strain, featuring overexpression of *davTBhisA* and *yahK* alongside *gabD2* deletion, achieved a titer of 52.1 g/L of 5-HV with a yield of 0.33 g/g glucose (Sohn et al., 2021).

Expanding on the established 5-HV biosynthesis system, further metabolic engineering efforts enabled the production of 1,5-PDO. A foundational strain capable of high-level 5-HV synthesis from glucose was first developed through targeted genome modifications, which included the deletion of genes responsible for by-product formation and the integration of core biosynthetic modules, eliminating the need for plasmid-based expression. Building on this platform, the 1,5-PDO biosynthesis pathway was systematically optimized through iterative module refinement. Two potential routes for converting 5-HV to 1,5-PDO were explored: (i) a CoA-independent pathway involving the direct reduction of 5-HV to 5-hydroxyvaleraldehyde, catalyzed by carboxylic acid reductase (CAR) and aldehyde reductase, and (ii) a CoA-dependent pathway requiring CoA transferase, CoA-acylating aldehyde dehydrogenase, and aldehyde reductase. Comparative analysis demonstrated that the CoA-independent pathway, catalyzed by CAR, was the more efficient route. A comprehensive screening of CAR enzymes, followed by protein engineering of *Mycobacterium avium subspecies paratuberculosis* MAP1040 CAR, yielded an optimized variant (M296E mutant) with enhanced activity. Additionally, a deep-learning-based analysis of aldehyde reductases identified *Gluconobacter oxydans* GOX1801 as the most effective enzyme for converting 5-hydroxyvaleraldehyde into 1,5-PDO. This integrated metabolic engineering approach established a robust and high-yielding bioprocess, achieving a titer of 43.4 g/L of 1,5-PDO from glucose (Sohn et al., 2024).

VL, a five-carbon lactam, has garnered significant interest as a monomer for nylon-5 and nylon-6,5 (Tiedemann et al., 2020). Its biosynthesis involves three key enzymes: DavB, DavA, and β-alanine CoA transferase (Act). In this pathway, DavB and DavA convert L-lysine into 5-AVA, which is subsequently cyclized into VL by Act. Recent advances in synthetic and systems metabolic engineering have enabled the development of *C. glutamicum* strains capable of high-level VL production. Given that L-lysine and 5-AVA serve as primary precursors, efforts have largely focused on optimizing precursor supply and minimizing by-product formation. A promising genome engineering tool, sRNA-induced gene regulation, has also been explored for enhancing VL biosynthesis. Unlike CRISPRi-Cas9, sRNA-based modulation imposes minimal metabolic burden while effectively controlling gene translation. The recently developed BHR-sRNA system was applied to *C. glutamicum* to identify key genetic targets for VL production (Cho et al., 2023). In flask cultivation, 12 *C. glutamicum* strains carrying different target-specific sRNAs were screened, revealing three knockdown targets—*gdh*, *hom*, and *icd*—that significantly enhanced VL production. Engineered strains carrying *act*, *davB*, and *davA*, in combination with *gdh*, *hom*, or *icd* knockdown, achieved VL titers of 5.08 g/L, 4.56 g/L, and 4.49 g/L, respectively. Further metabolic engineering strategies enabled the construction of high-performance VL-producing strains (Han & Lee, 2023). To prevent unwanted by-product formation, the *gabT* gene was deleted to eliminate GTA accumulation. Additionally, the 5-AVA importer gene *NCgl0464* was identified and overexpressed by replacing its native promoter with the strong H36 promoter using the *sacB* system, resulting in strain VL6 with an improved precursor pool. To enhance the flux from 5-AVA to VL, three copies of the *act* gene were integrated into the genome via the *sacB* system, yielding strain VL9. Finally, glucose uptake was optimized by upregulating genes involved in PTS-independent glucose transport, including *iolT1* and *iolT2* (inositol permeases) and *ppgk* (glucokinase), using *tuf*, *O6*, and *sod* promoters, respectively, leading to strain VL10. The final strain produced 9.68 g/L VL in flask cultivation, representing a fivefold improvement over the initial strain. In fed-batch fermentation, VL10 achieved a VL titer of 76.1 g/L, with a productivity of 0.99 g/L/h and a yield of 0.28 g/g. To further enhance production, dynamic regulation systems were explored to alleviate pathway bottlenecks (Zhao et al., 2023). A VL-responsive ChnR/Pb system was leveraged to create a positive feedback amplifier, which was further optimized into ChnR-B1/Pb-E1, exhibiting greater dynamic output and sensitivity. Implementation of this upregulation system in the final strain enabled VL production of 12.33 g/L in fed-batch fermentation, underscoring the potential of dynamic control strategies for improving industrial bioproduction.

## Conclusion

This review highlights the evolution of metabolic engineering, emphasizing the transition from traditional approaches to systems metabolic engineering, with a focus on strain development through the DBTL cycle. By integrating systems biology, synthetic biology, and evolutionary engineering, *C. glutamicum*—originally a well-established amino acid producer—has been transformed into a versatile microbial platform for synthesizing high-value chemicals. This progress highlights the potential of systems metabolic engineering as a sustainable and environmentally friendly alternative to conventional petrochemical processes. Notably, L-lysine, a key metabolite naturally produced by *C. glutamicum*, has expanded beyond its traditional industrial applications to serve as a precursor for valuable C5 chemicals such as 5-AVA, GTA, 5-HV, 1,5-PDO, and VL, demonstrating the growing impact of metabolic engineering advancements.

To establish predictable and robust microbial production platforms, it is essential to bridge the gap between systems biology-derived analytical insights and the experimental findings from synthetic biology while addressing the inherent uncertainties of evolutionary engineering. Future design strategies need to take a holistic approach, considering gene interactions, enzyme efficiencies, metabolic fluxes, protein complexes, and small-molecule dynamics. These efforts will contribute to refining the DBTL cycle and expanding its potential for more efficient strain development (Nakazawa et al., 2021). As integrated systems metabolic engineering continues to overcome the limitations of traditional metabolic engineering, it holds promise for resolving remaining technological challenges. With its pivotal role in enabling the sustainable and eco-friendly production of diverse biochemicals, systems metabolic engineering will remain at the forefront of industrial biotechnology, driving innovation toward a more sustainable future.

## Figures and Tables

**Table 1.** Value-added compounds produced by genomic engineering of *C. glutamicum*

Product	Host strain	Engineering technique	Titer (g/L)	Scale	References
Cadaverine	*C. glutamicum* PKC	√ Chromosomal integration of *E. coli* derived *ldcC* with a strong synthetic H30 promoter at the *lysE* site	103.78	Fed-batch	Kim et al. (2018)
	*C. glutamicum* PKC	√ Chromosomal integration of *H. alvei* derived *ldcC* with a strong synthetic H30 promoter at the *lysE* site	125	Fed-batch	Kim et al. (2019a)
	*C. glutamicum* KCTC 1857	√ Co-expression of *dr1558* and *cadA*	10.3	Fed-batch	Kang and Choi (2021)
	*C. glutamicum* KCTC 1857	√ Co-expression of *dr1558* and *ldcC*	25.1	Fed-batch	Kang and Choi (2022)
GTA	*C. glutamicum* KCTC 1857	√ Introduction of glutarate biosynthesis pathway by expressing *davTDBA* genes	24.5	Fed-batch	Kim et al. (2018)
		√ Gene modification of *davB* with an N-terminal His_6_-tag			
	*C. glutamicum* BE (*C. glutamicum* KCTC 12390BP)	√ Identification and expression of 11 target genes for increasing L-lysine supply through gene deletion/integration/substitution along with system-wide analyses	105.3	Fed-batch	Han et al. (2020)
		√ Overexpression of *ynfM*			
	*C. glutamicum* GRLys1	√ Introduction of glutarate biosynthesis pathway by expressing *ldcC*, *patDA*, *gabTD*^Stu^	25	Fed-batch	Pérez-García et al. (2018)
		√ Gene deletion of *sugR*, *ldhA*, *snaA*, *cgmA*, and *gdh*			
	*C. glutamicum* GSLA2 Δ*gdh*	√ Introduction of glutarate biosynthesis pathway by expressing *gltB*^E686Q^, *ldcC*, *patDA*, *tetA(Z)^Δ21bp^-gabTD^P134L^*	22.7	Fed-batch	Prell et al. (2021)
		√ Adaptive laboratory evolution			
5-AVA	*C. glutamicum* BE	√ Introduction of 5-AVA biosynthesis pathway by expressing *P. putida* derived *davB* and *davA*	33.1	Fed-batch	Shin et al. (2016)
		√ Overexpression of *davA* by fusing it with His_6_-Tag at its N-Terminal			
	*C. glutamicum* GRLys1	√ Introduction of 5-AVA biosynthesis pathway by expressing *E. coil* derived *ldcC*, *patA* and *patD*	5.1	Shake-flask	Jorge et al. (2017)
		√ Gene deletion of *sugR, ldhA, snaA, cgmA, and gabTDP*			
5-HV	*C. glutamicum* PKC	√ Introduction of 5-HV biosynthesis pathway by expressing *P. putida* derived *davTBA* and *E. coil* derived *yahK*	52.1	Fed-batch	Sohn et al. (2021)
		√ Gene deletion of *gabD*			
1,5-PDO	*C. glutamicum* PKC *ΔgabD2*	√ Introduction of 1,5-PDO biosynthesis pathway by expressing *M. marinum* derived carboxylic acid reductase (CAR) and *G. oxydans* derived GOX1801	43.4	Fed-batch	Sohn et al. (2024)
		√ Chromosomal integration of P_H30_*DavBHisA* expression cassette at the site of *lysE*			
		√ Enzyme engineering of CAR			
VL	*C. glutamicum* XT1	√ Introduction of valerolactam biosynthesis pathway by expressing *P. putida* derived *davBA* and *C. propionicum* derived *act*	12.33	Fed-batch	Zhao et al. (2023)
		√ Dynamic upregulation system using engineered ChnR-B1/Pb-E1 biosensor system			
	*C. glutamicum* GA16 Δ*gabT*	√ Gene expression down regulation of *gdh* using sRNA knock-down system	76.1	Fed-batch	Han and Lee (2023)
		√ Identification and engineering of 5-AVA transporter genes			
		√ Chromosomal integration of multiple copies of *act*			

## References

[b1-jm-2501021] Adkins J, Jordan J, Nielsen DR (2013). Engineering *Escherichia coli* for renewable production of the 5‐carbon polyamide building‐blocks 5‐aminovalerate and glutarate. Biotechnol Bioeng.

[b2-jm-2501021] Becker J, Zelder O, Hafner S, Schroder H, Wittmann C (2011). From zero to hero--design-based systems metabolic engineering of *Corynebacterium glutamicum* for L-lysine production. Metab Eng.

[b3-jm-2501021] Bendt AK, Burkovski A, Schaffer S, Bott M, Farwick M (2003). Towards a phosphoproteome map of *Corynebacterium glutamicum*. Proteomics.

[b4-jm-2501021] Binder S, Siedler S, Marienhagen J, Bott M, Eggeling L (2013). Recombineering in *Corynebacterium glutamicum* combined with optical nanosensors: a general strategy for fast producer strain generation. Nucleic Acids Res.

[b5-jm-2501021] Chae TU, Ko YS, Hwang KS, Lee SY (2017). Metabolic engineering of *Escherichia coli* for the production of four-, five- and six-carbon lactams. Metab Eng.

[b6-jm-2501021] Chai M, Deng C, Chen Q, Lu W, Liu Y (2021). Synthetic biology toolkits and metabolic engineering applied in *Corynebacterium glutamicum* for biomanufacturing. ACS Synth Biol.

[b7-jm-2501021] Chen W, Zhang Y, Yeo WS, Bae T, Ji Q (2017). Rapid and efficient genome editing in staphylococcus aureus by using an engineered CRISPR/cas9 system. J Am Chem Soc.

[b8-jm-2501021] Chen Z, Bommareddy RR, Frank D, Rappert S, Zeng AP (2014). Deregulation of feedback inhibition of phosphoenolpyruvate carboxylase for improved lysine production in *Corynebacterium glutamicum*. Appl Environ Microbiol.

[b9-jm-2501021] Cheng J, Chen P, Song A, Wang D, Wang Q (2018). Expanding lysine industry: Industrial biomanufacturing of lysine and its derivatives. J Ind Microbiol Biotechnol.

[b10-jm-2501021] Cho JS, Yang D, Prabowo CPS, Ghiffary MR, Han T (2023). Targeted and high-throughput gene knockdown in diverse bacteria using synthetic sRNAs. Nat Commun.

[b11-jm-2501021] Choi KR, Jang WD, Yang D, Cho JS, Park D (2019). Systems metabolic engineering strategies: Integrating systems and synthetic biology with metabolic engineering. Trends Biotechnol.

[b12-jm-2501021] Chuang HY, Hofree M, Ideker T (2010). A decade of systems biology. Annu Rev Cell Dev Biol.

[b13-jm-2501021] Cleto S, Jensen JV, Wendisch VF, Lu TK (2016). *Corynebacterium glutamicum* metabolic engineering with CRISPR interference (CRISPRi). ACS Synth Biol.

[b14-jm-2501021] Cremer J, Treptow C, Eggeling L, Sahm H (1988). Regulation of enzymes of lysine biosynthesis in *Corynebacterium glutamicum*. J Gen Microbiol.

[b15-jm-2501021] Demain AL (2000). Microbial biotechnology. Trends Biotechnol.

[b16-jm-2501021] Desai SC, Macrin AD, Senthilvelan T, Panda RC (2024). Identification of genes associated with accelerated biological ageing through computational analysis: a systematic review. Biotechnol Bioprocess Eng.

[b17-jm-2501021] Dragosits M, Mattanovich D (2013). Adaptive laboratory evolution -- principles and applications for biotechnology. Microb Cell Fact.

[b18-jm-2501021] Eggeling L, Bott M (2015). A giant market and a powerful metabolism: L-lysine provided by *Corynebacterium glutamicum*. Appl Microbiol Biotechnol.

[b19-jm-2501021] Eikmanns BJ, Eggeling L, Sahm H (1993). Molecular aspects of lysine, threonine, and isoleucine biosynthesis in *Corynebacterium glutamicum*. Antonie van Leeuwenhoek.

[b20-jm-2501021] Esvelt KM, Wang HH (2013). Genome‐scale engineering for systems and synthetic biology. Mol Syst Biol.

[b21-jm-2501021] Felix F, Letti LAJ, Vinicius de Melo Pereira G, Bonfim PGB, Soccol VT (2019). L-lysine production improvement: A review of the state of the art and patent landscape focusing on strain development and fermentation technologies. Crit Rev Biotechnol.

[b22-jm-2501021] Gilbert LA, Larson MH, Morsut L, Liu Z, Brar GA (2013). CRISPR-mediated modular RNA-guided regulation of transcription in eukaryotes. Cell.

[b23-jm-2501021] Godara A, Kao KC (2020). Adaptive laboratory evolution for growth coupled microbial production. World J Microbiol Biotechnol.

[b24-jm-2501021] Gonzalez-Ramos D, Gorter de Vries AR, Grijseels SS, van Berkum MC, Swinnen S (2016). A new laboratory evolution approach to select for constitutive acetic acid tolerance in saccharomyces cerevisiae and identification of causal mutations. Biotechnol Biofuels.

[b25-jm-2501021] Gorshkova NV, Lobanova JS, Tokmakova IL, Smirnov SV, Akhverdyan VZ (2018). Mu-driven transposition of recombinant mini-mu unit DNA in the *Corynebacterium glutamicum* chromosome. Appl Microbiol Biotechnol.

[b26-jm-2501021] Gupta A, Lee SG, Sung BH, Lee DH, Cho BK (2024). Advancing biofoundry development: strategies and challenges. Biotechnol Bioprocess Eng.

[b27-jm-2501021] Han T, Kim GB, Lee SY (2020). Glutaric acid production by systems metabolic engineering of an L-lysine-overproducing *Corynebacterium glutamicum*. Proc Natl Acad Sci USA.

[b28-jm-2501021] Han T, Lee SY (2023). Metabolic engineering of *Corynebacterium glutamicum* for the high-level production of valerolactam, a nylon-5 monomer. Metab Eng.

[b29-jm-2501021] Haupka C, Delépine B, Irla M, Heux S, Wendisch VF (2020). Flux enforcement for fermentative production of 5-aminovalerate and glutarate by *Corynebacterium glutamicum*. Catalysts.

[b30-jm-2501021] Hsu PD, Lander ES, Zhang F (2014). Development and applications of CRISPR-cas9 for genome engineering. Cell.

[b31-jm-2501021] Huang H, Chai C, Li N, Rowe P, Minton NP (2016). CRISPR/cas9-based efficient genome editing in *Clostridium ljungdahlii*, an autotrophic gas-fermenting bacterium. ACS Synth Biol.

[b32-jm-2501021] Hwang Y, Noh MH, Jung GY (2024). Recent advancements in flavonoid production through engineering microbial systems. Biotechnol Bioprocess Eng.

[b33-jm-2501021] Ikeda M, Nakagawa S (2003). The *Corynebacterium glutamicum* genome: features and impacts on biotechnological processes. Appl Microbiol Biotechnol.

[b34-jm-2501021] Inui M, Tsuge Y, Suzuki N, Vertes AA, Yukawa H (2005). Isolation and characterization of a native composite transposon, tn 14751, carrying 17.4 kilobases of *Corynebacterium glutamicum* chromosomal DNA. Appl Environ Microbiol.

[b35-jm-2501021] Jäger W, Schafer A, Puhler A, Labes G, Wohlleben W (1992). Expression of the *Bacillus subtilis*
*sacB* gene leads to sucrose sensitivity in the gram-positive bacterium *Corynebacterium glutamicum* but not in Streptomyces lividans. J Bacteriol.

[b36-jm-2501021] Jorge JM, Pérez-García F, Wendisch VF (2017). A new metabolic route for the fermentative production of 5-aminovalerate from glucose and alternative carbon sources. Bioresour Technol.

[b37-jm-2501021] Jung HJ, Shin Y, Hwang JH, Shin N, Kim HJ (2024). Establishment of an optimized electroporation method for *Halomonas* sp. YK44 and its application in the coproduction of PHB and isobutanol. Biotechnol Bioprocess Eng.

[b38-jm-2501021] Kang S, Choi J (2021). Enhanced cadaverine production by recombinant *Corynebacterium glutamicum* with a heterologous dr1558 regulator at low pH condition. Process Biochem.

[b39-jm-2501021] Kang S, Choi J (2022). Production of cadaverine in recombinant *Corynebacterium glutamicum* overexpressing lysine decarboxylase (*ldcC*) and response regulator dr1558. Appl Biochem Biotechnol.

[b40-jm-2501021] Kiefer P, Heinzle E, Zelder O, Wittmann C (2004). Comparative metabolic flux analysis of lysine-producing *Corynebacterium glutamicum* cultured on glucose or fructose. Appl Environ Microbiol.

[b41-jm-2501021] Kim HT, Baritugo K, Hyun SM, Khang TU, Sohn YJ (2019a). Development of metabolically engineered *Corynebacterium glutamicum* for enhanced production of cadaverine and its use for the synthesis of bio-polyamide 510. ACS Sustain Chem Eng.

[b42-jm-2501021] Kim HT, Baritugo K, Oh YH, Hyun SM, Khang TU (2018). Metabolic engineering of *Corynebacterium glutamicum* for the high-level production of cadaverine that can be used for the synthesis of biopolyamide 510. ACS Sustain Chem Eng.

[b43-jm-2501021] Kim HT, Khang TU, Baritugo K, Hyun SM, Kang KH (2019b). Metabolic engineering of *Corynebacterium glutamicum* for the production of glutaric acid, a C5 dicarboxylic acid platform chemical. Metab Eng.

[b44-jm-2501021] Kim GY, Kim J, Park G, Kim HJ, Yang J (2023). Synthetic biology tools for engineering *Corynebacterium glutamicum*. Comput Struct Biotechnol J.

[b45-jm-2501021] Kim T, Ko M, Rha E, Kim H, Lee H (2024). Microfluidics-driven high-throughput phenotyping and screening in synthetic biology: from single cells to cell-free systems. Biotechnol. Bioprocess Eng.

[b46-jm-2501021] Kind S, Jeong WK, Schroder H (2010). Systems-wide metabolic pathway engineering in *Corynebacterium glutamicum* for bio-based production of diaminopentane. Metab Eng.

[b47-jm-2501021] Kind S, Neubauer S, Becker J, Yamamoto M, Völkert M (2014). From zero to hero-production of bio-based nylon from renewable resources using engineered *Corynebacterium glutamicum*. Metab Eng.

[b48-jm-2501021] Kromer JO, Sorgenfrei O, Klopprogge K, Heinzle E, Wittmann C (2004). In-depth profiling of lysine-producing *Corynebacterium glutamicum* by combined analysis of the transcriptome, metabolome, and fluxome. J Bacteriol.

[b49-jm-2501021] Larson MH, Gilbert LA, Wang X, Lim WA, Weissman JS (2013). CRISPR interference (CRISPRi) for sequence-specific control of gene expression. Nat Protoc.

[b50-jm-2501021] Lee SY, Kim HU, Chae TU, Cho JS, Kim JW (2019). A comprehensive metabolic map for production of bio-based chemicals. Nat Catal.

[b51-jm-2501021] Lee JW, Kim TY, Jang YS, Choi S, Lee SY (2011). Systems metabolic engineering for chemicals and materials. Trends Biotechnol.

[b52-jm-2501021] Lee WH, Pathanibul P, Quarterman J, Jo JH, Han NS (2012). Whole cell biosynthesis of a functional oligosaccharide, 2'-fucosyllactose, using engineered *Escherichia coli*. Microb Cell Fact.

[b53-jm-2501021] Lee J, Shin H, Lee KH, Lee H, Lee G (2024). Component analysis and utilization strategy of brown macroalgae as promising feedstock for sugar platform-based marine biorefinery. Biotechnol. Bioprocess Eng.

[b54-jm-2501021] Li M, Li D, Huang Y, Liu M, Wang H (2014). Improving the secretion of cadaverine in Corynebacterium glutamicum by cadaverine–lysine antiporter. J Ind Microbiol Biotechnol.

[b55-jm-2501021] Lindner SN, Niederholtmeyer H, Schmitz K, Schoberth SM, Wendisch VF (2010). Polyphosphate/ATP-dependent NAD kinase of *Corynebacterium glutamicum*: biochemical properties and impact of *ppnK* overexpression on lysine production. Appl Microbiol Biotechnol.

[b56-jm-2501021] Liu Z, Liu J, Zhang F, Zhang W (2024a). Modifying *Corynebacterium glutamicum* by metabolic engineering for efficient synthesis of L-lysine. Syst Microbiol Biomanuf.

[b57-jm-2501021] Liu J, Ou Y, Xu JZ, Rao ZM, Zhang WG (2023). L-lysine production by systems metabolic engineering of an NADPH auto-regulated *Corynebacterium glutamicum*. Bioresour Technol.

[b58-jm-2501021] Liu WL, Wang HY, Li M, Xu GT, Xu JZ (2024b). The 279th residue of aspartate kinase in *Corynebacterium glutamicum* is important for relieving the feedback inhibition by L-lysine and L-threonine. Mol Catal.

[b59-jm-2501021] Liu J, Xu JZ, Rao ZM, Zhang WG (2022). Industrial production of L-lysine in *Corynebacterium glutamicum*: Progress and prospects. Microbiol Res.

[b60-jm-2501021] Liu C, Zhang L, Liu H, Cheng K (2017). Delivery strategies of the CRISPR-cas9 gene-editing system for therapeutic applications. J Control Release.

[b61-jm-2501021] Luntz MG, Zhdanova NI, Bourd GI (1986). Transport and excretion of L-lysine in *Corynebacterium glutamicum*. Microbiology.

[b62-jm-2501021] Mans R, Daran JG, Pronk JT (2018). Under pressure: Evolutionary engineering of yeast strains for improved performance in fuels and chemicals production. Curr Opin Biotechnol.

[b63-jm-2501021] Mimitsuka T, Sawai H, Hatsu M, Yamada K (2007). Metabolic engineering of *Corynebacterium glutamicum* for cadaverine fermentation. Biosci Biotechnol Biochem.

[b64-jm-2501021] Mormann S, Lömker A, Rückert C, Gaigalat L, Tauch A (2006). Random mutagenesis in *Corynebacterium glutamicum* ATCC 13032 using an IS 6100-based transposon vector identified the last unknown gene in the histidine biosynthesis pathway. BMC Genom.

[b65-jm-2501021] Nakazawa S, Imaichi O, Kogure T, Kubota T, Toyoda K (2021). History-driven genetic modification design technique using a domain-specific lexical model for the acceleration of DBTL cycles for microbial cell factories. ACS Synth Biol.

[b66-jm-2501021] Naseri G, Koffas MA (2020). Application of combinatorial optimization strategies in synthetic biology. Nat Commun.

[b67-jm-2501021] Oh YH, Choi JW, Kim EY, Song BK, Jeong KJ (2015). Construction of synthetic promoter-based expression cassettes for the production of cadaverine in recombinant *Corynebacterium glutamicum*. Appl Biochem Biotechnol.

[b68-jm-2501021] Ohnishi J, Katahira R, Mitsuhashi S, Kakita S, Ikeda M (2005). A novel *gnd* mutation leading to increased L-lysine production in *Corynebacterium glutamicum*. FEMS Microbiol Lett.

[b69-jm-2501021] Ohnishi J, Mitsuhashi S, Hayashi M, Ando S, Yokoi H (2002). A novel methodology employing *Corynebacterium glutamicum* genome information to generate a new L-lysine-producing mutant. Appl Microbiol Biotechnol.

[b70-jm-2501021] Park SJ, Kim EY, Noh W, Park HM, Oh YH (2013). Metabolic engineering of *Escherichia coli* for the production of 5-aminovalerate and glutarate as C5 platform chemicals. Metab Eng.

[b71-jm-2501021] Paquet D, Kwart D, Chen A, Sproul A, Jacob S (2016). Efficient introduction of specific homozygous and heterozygous mutations using CRISPR/cas9. Nature.

[b72-jm-2501021] Peng F, Wang X, Sun Y, Dong G, Yang Y (2017). Efficient gene editing in *Corynebacterium glutamicum* using the CRISPR/cas9 system. Microb Cell Fact.

[b73-jm-2501021] Pérez-García F, Jorge JM, Dreyszas A, Risse JM, Wendisch VF (2018). Efficient production of the dicarboxylic acid glutarate by *Corynebacterium glutamicum* via a novel synthetic pathway. Front Microbiol.

[b74-jm-2501021] Plachý J, Ulbert S, Pelechová J, Krfmphanzl V (1985). Fermentation production of L-homoserine by *Corynebacterium* sp. and its possible use in the preparation of threonine and lysine. Folia Microbiol.

[b75-jm-2501021] Prell C, Busche T, Ruckert C, Nolte L, Brandenbusch C (2021). Adaptive laboratory evolution accelerated glutarate production by *Corynebacterium glutamicum*. Microb Cell Fact.

[b76-jm-2501021] Quast K, Bathe B, Pühler A, Kalinowski J (1999). The *Corynebacterium glutamicum* insertion sequence ISCg2 prefers conserved target sequences located adjacent to genes involved in aspartate and glutamate metabolism. Mol Gen Genet.

[b77-jm-2501021] Richardson CD, Ray GJ, DeWitt MA, Curie GL, Corn JE (2016). Enhancing homology-directed genome editing by catalytically active and inactive CRISPR-cas9 using asymmetric donor DNA. Nat Biotechnol.

[b78-jm-2501021] Rohles CM, Giesselmann G, Kohlstedt M, Wittmann C, Becker J (2016). Systems metabolic engineering of *Corynebacterium glutamicum* for the production of the carbon-5 platform chemicals 5-aminovalerate and glutarate. Microb Cell Fact.

[b79-jm-2501021] Rohles CM, Gläser L, Kohlstedt M, Gießelmann G, Pearson S (2018). A bio-based route to the carbon-5 chemical glutaric acid and to bionylon-6, 5 using metabolically engineered *Corynebacterium glutamicum*. Green Chem.

[b80-jm-2501021] Rohles C, Pauli S, Gießelmann G, Kohlstedt M, Becker J (2022). Systems metabolic engineering of *Corynebacterium glutamicum* eliminates all by-products for selective and high-yield production of the platform chemical 5-aminovalerate. Metab Eng.

[b81-jm-2501021] Sandberg TE, Salazar MJ, Weng LL, Palsson BO, Feist AM (2019). The emergence of adaptive laboratory evolution as an efficient tool for biological discovery and industrial biotechnology. Metab Eng.

[b82-jm-2501021] Schäfer A, Tauch A, Jager W, Kalinowski J, Thierbach G (1994). Small mobilizable multi-purpose cloning vectors derived from the *Escherichia coli* plasmids pK18 and pK19: Selection of defined deletions in the chromosome of *Corynebacterium glutamicum*. Gene.

[b83-jm-2501021] Schrumpf B, Eggeling L, Sahm H (1992). Isolation and prominent characteristics of an L-lysine hyperproducing strain of *Corynebacterium glutamicum*. Appl Environ Microbiol.

[b84-jm-2501021] Schrumpf B, Schwarzer A, Kalinowski J, Puhler A, Eggeling L (1991). A functionally split pathway for lysine synthesis in Corynebacterium glutamicium. J Bacteriol.

[b85-jm-2501021] Shah AH, Hameed A, Ahmad S, Khan GM (2002). Optimization of culture conditions for L-lysine fermentation by *Corynebacterium glutamicum*. Biol Sci.

[b86-jm-2501021] Shalem O, Sanjana NE, Hartenian E, Shi X, Scott DA (2014). Genome-scale CRISPR-cas9 knockout screening in human cells. Science.

[b87-jm-2501021] Shin JH, Park SH, Oh YH, Choi JW, Lee MH (2016). Metabolic engineering of *Corynebacterium glutamicum* for enhanced production of 5-aminovaleric acid. Microb Cell Fact.

[b88-jm-2501021] Sohn YJ, Hwang SY, Lee H, Jeon S, Park JY (2024). Metabolic engineering of *Corynebacterium glutamicum* for high-level production of 1,5-pentanediol, a C5 diol platform chemical. Adv Sci.

[b89-jm-2501021] Sohn YJ, Kang M, Baritugo K, Son J, Kang KH (2021). Fermentative high-level production of 5-hydroxyvaleric acid by metabolically engineered *Corynebacterium glutamicum*. ACS Sustain Chem Eng.

[b90-jm-2501021] Sohn YJ, Kang M, Ryu M, Lee S, Kang KH (2022). Development of a bio-chemical route to C5 plasticizer synthesis using glutaric acid produced by metabolically engineered *Corynebacterium glutamicum*. Green Chem.

[b91-jm-2501021] Son J, Sohn YJ, Baritugo K, Jo SY, Song HM (2023). Recent advances in microbial production of diamines, aminocarboxylic acids, and diacids as potential platform chemicals and bio-based polyamides monomers. Biotechnol Adv.

[b92-jm-2501021] Stella RG, Gertzen CG, Smits SH, Gätgens C, Polen T (2021). Biosensor-based growth-coupling and spatial separation as an evolution strategy to improve small molecule production of *Corynebacterium glutamicum*. Metab Eng.

[b93-jm-2501021] Stella RG, Wiechert J, Noack S, Frunzke J (2019). Evolutionary engineering of *Corynebacterium glutamicum*. Biotechnol J.

[b94-jm-2501021] Suzuki N, Okai N, Nonaka H, Tsuge Y, Inui M (2006). High-throughput transposon mutagenesis of *Corynebacterium glutamicum* and construction of a single-gene disruptant mutant library. Appl Environ Microbiol.

[b95-jm-2501021] Suzuki N, Tsuge Y, Inui M, Yukawa H (2005). Cre/*loxP*-mediated deletion system for large genome rearrangements in *Corynebacterium glutamicum*. Appl Microbiol Biotechnol.

[b96-jm-2501021] Tan Y, Xu D, Li Y, Wang X (2012). Construction of a novel *sacB*-based system for marker-free gene deletion in *Corynebacterium glutamicum*. Plasmid.

[b97-jm-2501021] Tauch A, Kassing F, Kalinowski J, Pühler A (1995). The Corynebacterium xerosis composite transposon Tn5432 consists of two identical insertion sequences, designated IS1249, flanking the erythromycin resistance gene *ermCX*. Plasmid.

[b98-jm-2501021] Tosaka O, Koichi T, Hirose Y (1978). Production of L-lysine by leucine auxotrophs derived from AEC resistant mutant of *Brevibacterium lactofermentum*. Agric Biol Chem.

[b99-jm-2501021] Vertès AA, Inui M, Kobayashi M, Kurusu Y, Yukawa H (1994). Isolation and characterization of IS31831, a transposable element from *Corynebacterium glutamicum*. Mol Microbiol.

[b100-jm-2501021] von Tiedemann P, Anwar S, Kemmer‐Jonas U, Asadi K, Frey H (2020). Synthesis and solution processing of nylon‐5 ferroelectric thin films: The renaissance of odd‐nylons?. Macromol Chem Phys.

[b101-jm-2501021] Vrljic M, Sahm H, Eggeling L (1996). A new type of transporter with a new type of cellular function: L-lysine export from *Corynebacterium glutamicum*. Mol Microbiol.

[b102-jm-2501021] Wang G, Li Q, Zhang Z, Yin X, Wang B (2023). Recent progress in adaptive laboratory evolution of industrial microorganisms. J Ind Microbiol Biotechnol.

[b103-jm-2501021] Wen J, Sun W, Leng G, Li D, Feng C (2024). Enhanced fermentative γ-aminobutyric acid production by a metabolic engineered *Corynebacterium glutamicum*. Biotechnol Bioprocess Eng.

[b104-jm-2501021] Wu W, Zhang Y, Liu D, Chen Z (2019). Efficient mining of natural NADH-utilizing dehydrogenases enables systematic cofactor engineering of lysine synthesis pathway of *Corynebacterium glutamicum*. Metab Eng.

[b105-jm-2501021] Xu JZ, Liu WL, Zhang WG, Lu C (2024). Creation of a NADH-dependent *meso*-diaminopimelate dehydrogenase by site-saturation mutagenesis for producing L-lysine in *Corynebacterium glutamicum*. Mol Catal.

[b106-jm-2501021] Zerbini F, Zanella I, Fraccascia D, Konig E, Irene C (2017). Large scale validation of an efficient CRISPR/Cas-based multi gene editing protocol in *Escherichia coli*. Microb Cell Fact.

[b107-jm-2501021] Zhang Y, Cai J, Shang X, Wang B, Liu S (2017). A new genome-scale metabolic model of *Corynebacterium glutamicum* and its application. Biotechnol Biofuels.

[b108-jm-2501021] Zhao X, Wu Y, Feng T, Shen J, Lu H (2023). Dynamic upregulation of the rate-limiting enzyme for valerolactam biosynthesis in *Corynebacterium glutamicum*. Metab Eng.

[b109-jm-2501021] Zhao G, Zhang D, Zhou B, Li Z, Liu G (2024). Fine-regulating the carbon flux of L-isoleucine producing *Corynebacterium glutamicum* WM001 for efficient L-threonine production. ACS Synth Biol.

